# Supplementary figures and images for: Geographic barriers to care persist at the community healthcare level: Evidence from rural Madagascar
Source: PLOS Glob Public Health. 2022 Dec 27;2(12):e0001028. doi: 10.1371/journal.pgph.0001028 (PMC10022327; doi:10.1371/journal.pgph.0001028)

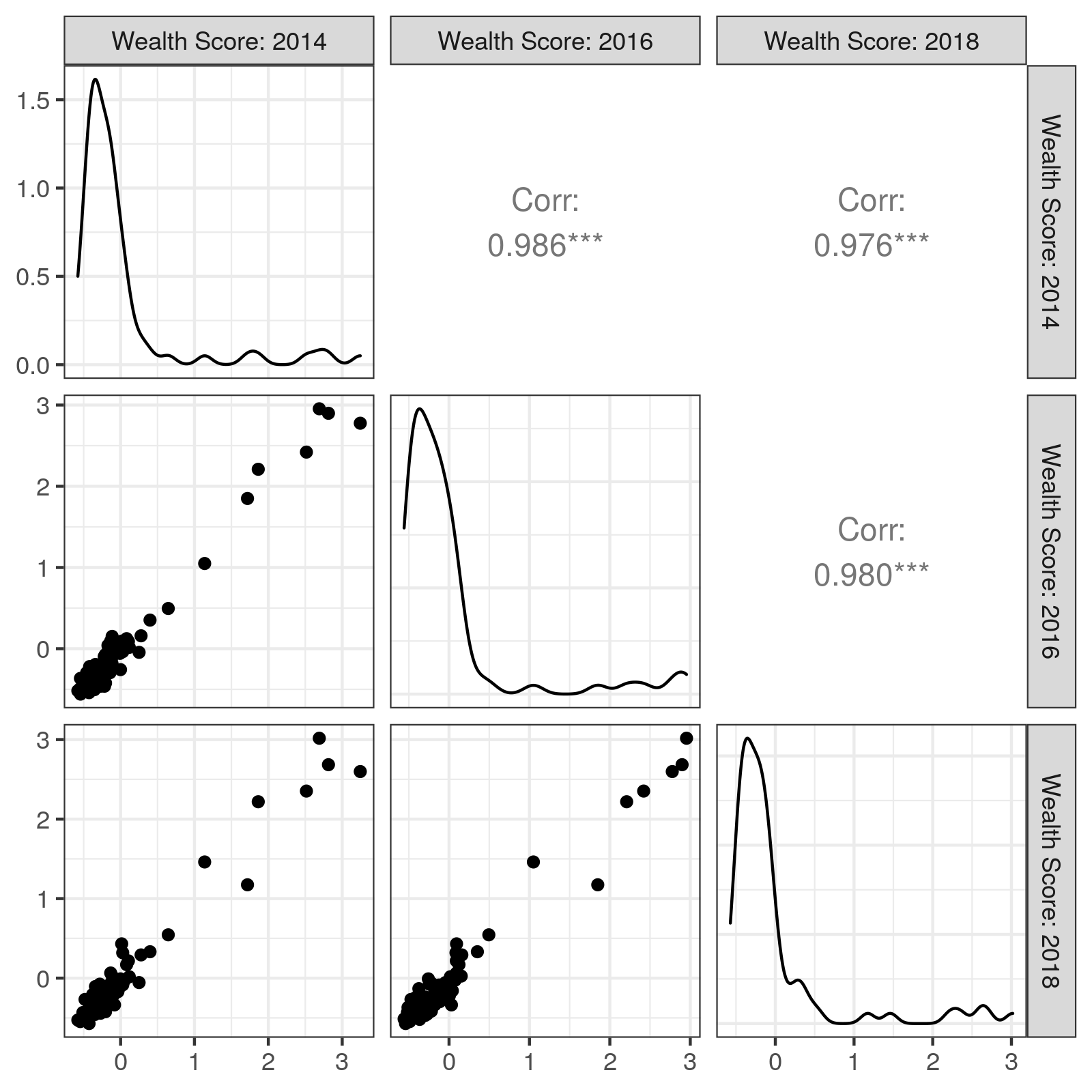

Supplement: S1 Fig — Correlation plots of wealth scores for the 80 clusters in the longitudinal cohort across the three sample years. Wealth scores are the mean of households in that cluster. (TIF) [file pgph.0001028.s001.tif]

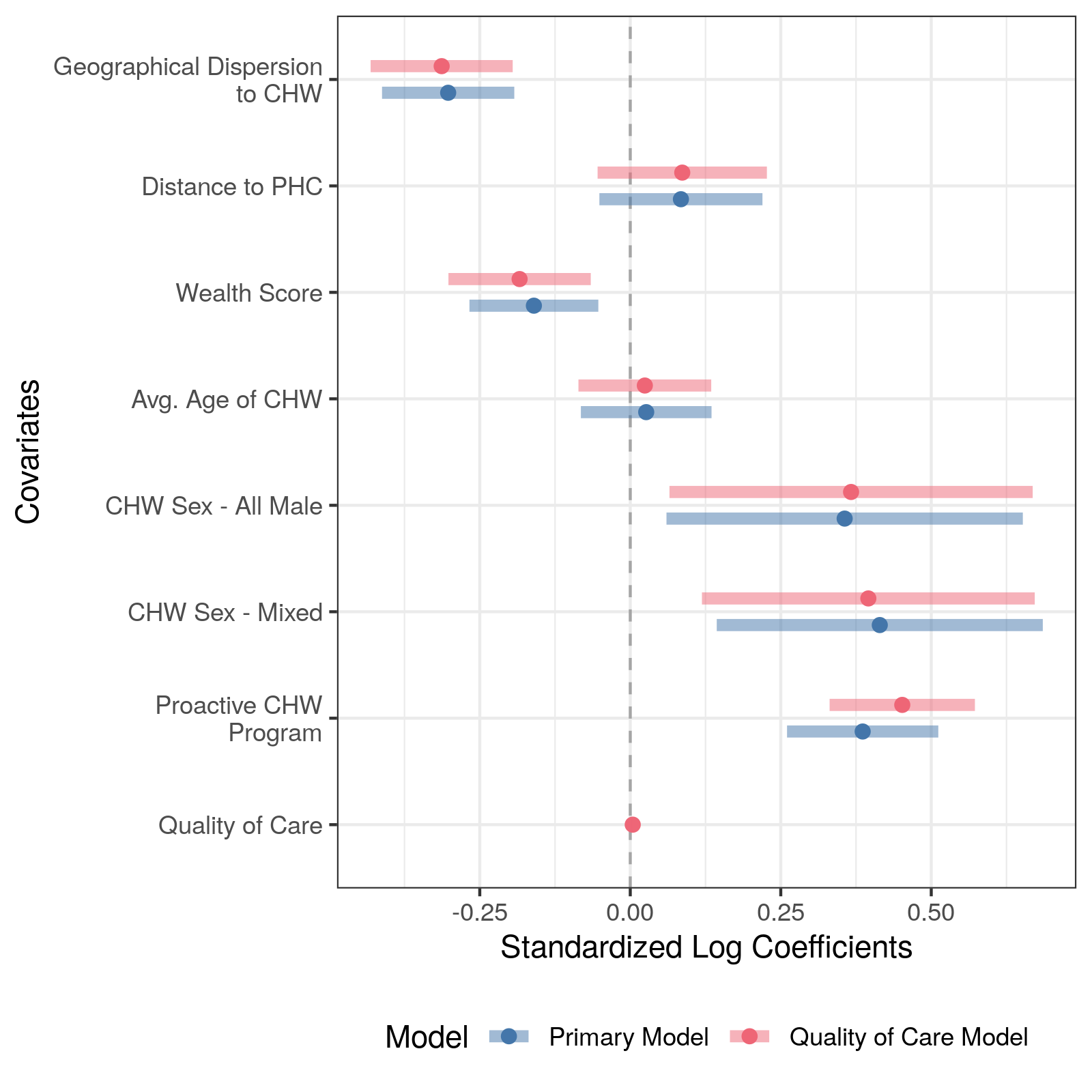

Supplement: S2 Fig — Comparison of standardized log-coefficients from the primary model and the supplemental ‘quality of care’ model on a subset of the data. Points represent mean coefficient and error bars 95% CIs. (TIF) [file pgph.0001028.s002.tif]

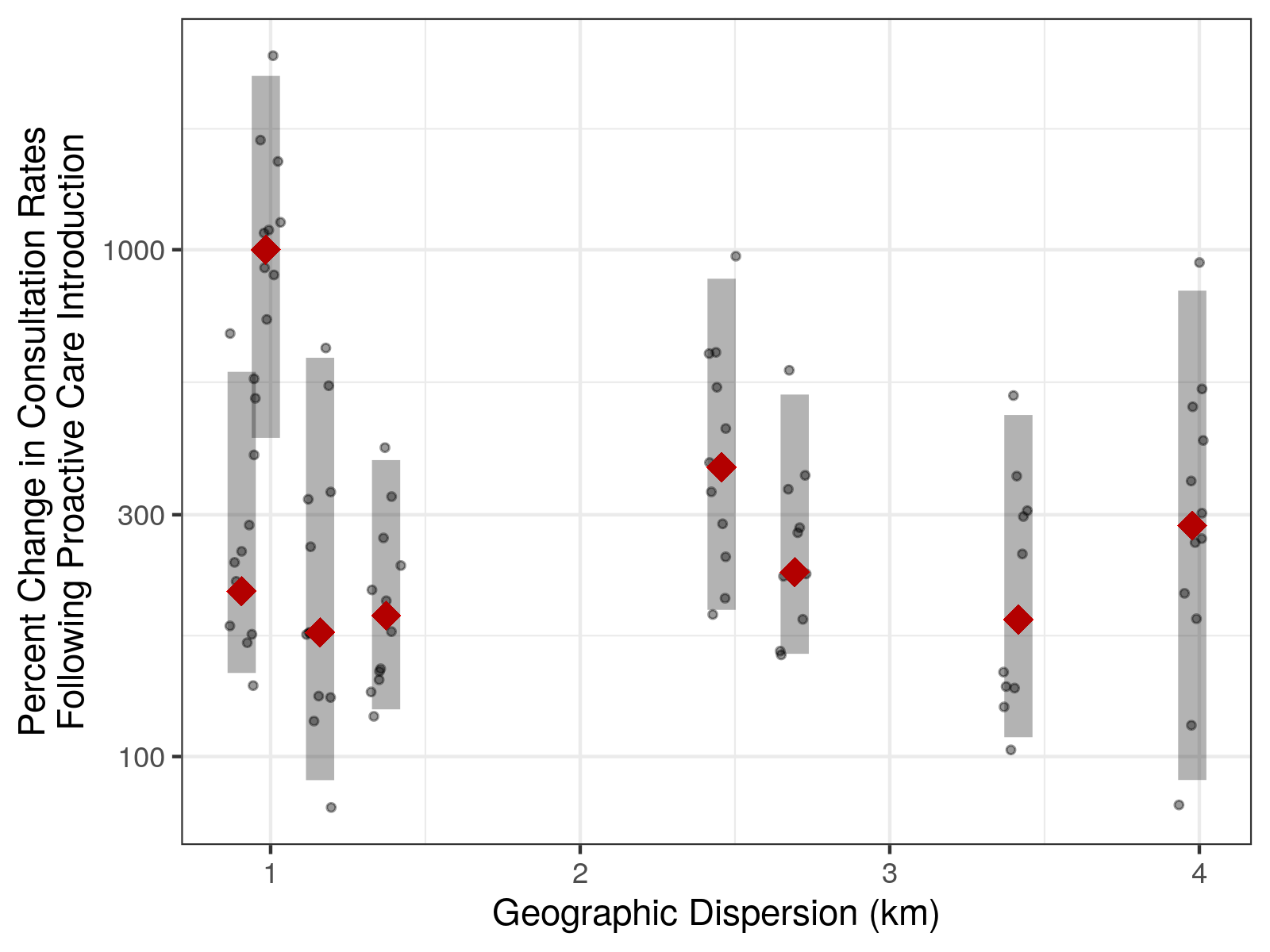

Supplement: S3 Fig — Points represent the percent change in consultation rates for each month of the year by comparing pre-proactive care (2017 and 2018) and post-proactive care (2020) for the eight fokontany in Ranomafana commune. Red diamonds represent the median per fokontany and shaded bars represent the 95% CI. (TIF) [file pgph.0001028.s003.tif]
