# Supplementary material for: Geographic barriers to care persist at the community healthcare level: Evidence from rural Madagascar
Source: PLOS Glob Public Health. 2022 Dec 27;2(12):e0001028. doi: 10.1371/journal.pgph.0001028 (PMC10022327; doi:10.1371/journal.pgph.0001028)
Supplement: S1 Table — (DOCX) [file pgph.0001028.s004.docx]

| **Area** | **Number of Fokontany** |
| --- | --- |
| 1.94 - 4.98 km^2^ | 14 |
| 5.05 - 9.98 km^2^ | 37 |
| 10.04 - 19.99 km^2^ | 72 |
| 20.75 - 39.95 km^2^ | 55 |
| 40.41 - 138.95 km^2^ | 17 |

**S1 Table.** Distribution of fokontany sizes in Ifanadiana district.
