# Supplementary material for: Geographic barriers to care persist at the community healthcare level: Evidence from rural Madagascar
Source: PLOS Glob Public Health. 2022 Dec 27;2(12):e0001028. doi: 10.1371/journal.pgph.0001028 (PMC10022327; doi:10.1371/journal.pgph.0001028)
Supplement: S1 File — (DOCX) [file pgph.0001028.s006.docx]

**SUPPLEMENTAL MATERIALS - REFLEXIVITY STATEMENT**

**Geographic barriers to care persist even at the community healthcare level: evidence from rural Madagascar**

Michelle V Evans*, Tanjona Andréambeloson*, Mauricianot Randriamihaja, Felana Ihantamalala, Laura Cordier, Giovanna Cowley, Karen Finnegan, Feno Hanitriniaina, Ann Miller, Lanto Marovavy Ralantomalala, Andry Randriamahasoa, Bénédicte Razafinjato^2^ Emeline Razanahanitriniaina,Rado JL Rakotonanahary, Isaïe Jules Andriamiandra, Matthew H Bonds, Andres Garchitorena

* Contributed equally

Corresponding Author: MVE, mv.evans.phd@gmail.com

The production of knowledge takes place within the existing socio-political landscape, and therefore easily reproduces existing power dynamics across identities including but not limited to gender, race, nationality, and class^[[1]](#footnote-2)^. Without explicit consideration of these dynamics and purposeful action to address inequalities, research is at best extractive and at worst harmful to the communities that are the subject of research ^[[2]](#footnote-3)^. This is a particular risk in this study, which includes authors from high income countries (HIC) and low-and-middle-income countries (LMIC) and whose positions at academic, public health, and development institutions imply different agendas. To encourage our own purposeful consideration of these power dynamics within the context of this project and be transparent to the broader research and public health community, we chose to record our reflections on equitable authorship in a formal reflexivity statement, following Morton et al.^[[3]](#footnote-4)^:

**Study conceptualization**

*How does this study address local research and policy priorities?*

This study focuses on Ifanadiana District in southeastern Madagascar, which has been established as a model district for universal health coverage by the government of Madagascar. One focus of this government intervention is the strengthening of the community healthcare system. By investigating the existence of a geographic barrier to community healthcare, this study identifies a potential obstacle to the goal of universal health coverage established by national policy and proposes several solutions.

*How were local researchers involved in study design?*

One data source for this study, the IHOPE longitudinal survey, was designed and conducted through a collaboration with the Madagascar Institute of Statistics, a national research institute. In addition, local researchers and public health stakeholders were involved in the identification of the research question for this specific project. Geographical access was chosen as a focus of this study in response to needs of community health stakeholders at the Madagascar Ministry of Public Health and the health system strengthening non-governmental organization Pivot to better understand current barriers to healthcare access.

However, through discussions with our research time while drafting this reflection statement, we noticed that there were many perspectives missing from the conceptualization step, notably local communities who do not identify as researchers, as well as co-authors who joined the project later in the process. We are currently evaluating our research approach to improve this in the future by explicitly scheduling more open discussion with a wider group of stakeholders during the conceptualization phase. In addition, we hope to adapt our research process to be less linear, scheduling feedback on all stages throughout the project and reviewing past decisions when necessary.

**Research management**

*How has funding been used to support the local research team(s)?*

This project used data that had already been collected from past projects and any financial support was used for researchers’ salaries. This included salaries for M Evans, an American post-doc based at a French institute, T Andréambeloson, a Malagasy doctoral student based at a Malagasy institute, and M Randriamihaja, a Malagasy research assistant working for Pivot, an international NGO working in Ifanadiana, Madagascar.

**Data acquisition and analysis**

*How are research staff who conducted data collection acknowledged?*

Data collection was conducted by staff at Harvard University, the Madagascar Ministry of Public Health (MMoPH), and Pivot. Project supervisors from Harvard, MMoPH, and Pivot are therefore included as co-authors on this project. In addition, two researchers from Pivot, F Ihantamalala and M Randriamihaja, created the geographic datasets and are included as co-authors.

*How have members of the research partnership been provided with access to study data?*

Geographic data is available as an open-source dataset on OpenStreetMap and via an online distance calculation tool developed by F Ihantamalala (<https://research.pivot-dashboard.org/>). The consultation data is available to Pivot and Madagascar Ministry of Public Health staff via an internal data management system.

*How were data used to develop analytical skills within the partnership?*

The statistical analyses were conducted as part of T Andréambeloson’s doctoral training program, during which she was supervised and trained by A Garchitorena. The geographical analyses were conducted by F Ihantamalala, an established geographic researcher, and M Randriamihaja, a research assistant, who was supervised by F Ihantamalala and A Garchitorena. Through this project, M Evans and M Randiramihaja also developed their own expertise in geospatial analysis techniques, working together to share knowledge across axes of academic seniority (post-doc and research assistant) and nationality (e.g. a North-South partnership).

**Data interpretation**

*How have research partners collaborated in interpreting study data?*

Data interpretation was conducted primarily by M Evans, T Andréambeloson, and A Garchitorena. However, preliminary research findings were shared broadly with Pivot staff through multiple meetings with different departments. Staff offered valuable field-based context and insights to ensure data was being interpreted properly within the context of Ifanadiana’s health system. They also provided programmatic and social context for results, which are described in the main text.

**Drafting and revising for intellectual content**

*How were research partners supported to develop writing skills?*

The majority of the manuscript was drafted by M Evans and A Garchitorena. All other co-authors were involved in the revision of the draft and validation of the final manuscript, but no explicit support was provided for developing partners’ writing skills.

*How will research products be shared to address local needs?*

Products of this research have been shared internally with Pivot staff via formal presentations and informal discussions, so that it can be included in decision making regarding community health interventions. Peer-reviewed research products will be shared by distributing the manuscript and summaries (translated into English, French, and Malagasy) to collaborators at local public health and research institutes. In addition, we have created a freely available online application that communicates the results of this research and allows community health supervisors to compare potential CHW site locations (https://research.pivot-dashboard.org). Training on how to use this application was provided by M Evans and M Randiramihaja to other Pivot staff.

**Authorship**

*How is the leadership, contribution and ownership of this work by LMIC researchers recognized within the authorship?*

Our author list includes researchers and staff who supervised teams involved in data collection of this project, as well as those who conducted specific data collection or analyses individually. This includes LMIC researchers who supervised collection of the health system and geographic data. Additionally, we use co-first authorship to convey shared ownership of this work between M Evans and T Andréambeloson, who were the primary researchers on this project, and who are from the USA and Madagascar, respectively.

*How have early career researchers across the partnership been included within the authorship team?*

The majority of this work was conducted by early career researchers, and this is reflected in the authorship team, which includes research assistants, doctoral students, and post-doctoral researchers, who are all listed at the beginning of the authorship list.

*How has gender balance been addressed within the authorship?*

The majority of co-authors are women, including the two first co-authors, and this gender ratio remains whether considering authors from HICs or LMICs. However, the two senior authors are men. Given the large collaborative nature of this project, however, the senior authors’ role in decision making was not disproportionately larger than other authors, and, upon reflecting on our scientific process, we believe there was equality among genders in decision making.

**Training**

*How has the project contributed to training of LMIC researchers?*

This project primarily trained two LMIC researchers. T Amdreambeloson worked on this project as part of a postgraduate research internship, and learned statistical analyses and the R programming language via this project. M Randriamihaja worked on this project as part of his position as a research assistant at Pivot, and gained skills implementing geographic routing algorithms and building Shiny applications through this work.

**Infrastructure**

*How has the project contributed to improvements in local infrastructure?*

This project was relatively small in scope with no project funding, and so did not involve the installation or improvement of infrastructure, outside of the creation of the online dashboard. However, we believe the results of this work will be useful to local stakeholders for improving the existing community health system by providing evidence of current barriers and support for the introduction of alternative community health interventions.

**Governance**

*What safeguarding procedures were used to protect local study participants and researchers?*

All individually identifiable data was collected following ethical procedures approved by the Madagascar National Ethics Committee and the Harvard Medical School IRB. In addition, IHOPE surveys were conducted by Malagasy researchers trained in ethical survey methods and consent was given by all participants. The Pivot-MMoPH partnership provided health system strengthening support to the community health sites from which participant data was collected for this study. Local research work was primarily office-based, and strict COVID-19 measures were in place at the Pivot ad IRD offices in Madagascar during the duration of each wave of the COVID-19 epidemic. In addition, the manuscript is approved by all co-authors, with a special focus on ensuring the language used is approved by MMoPH collaborators.

1. . Bhakuni, H., and S. Abimbola. 2021. Epistemic injustice in academic global health. The Lancet Global Health 9:e1465–e1470. [↑](#footnote-ref-2)
2. . Abimbola, S., S. Asthana, C. Montenegro, R. R. Guinto, D. T. Jumbam, L. Louskieter, K. M. Kabubei, S. Munshi, K. Muraya, F. Okumu, S. Saha, D. Saluja, and M. Pai. 2021. Addressing power asymmetries in global health: Imperatives in the wake of the COVID-19 pandemic. PLOS Medicine 18:e1003604. [↑](#footnote-ref-3)
3. . Morton, B., A. Vercueil, R. Masekela, E. Heinz, L. Reimer, S. Saleh, C. Kalinga, M. Seekles, B. Biccard, J. Chakaya, S. Abimbola, A. Obasi, and N. Oriyo. 2022. Consensus statement on measures to promote equitable authorship in the publication of research from international partnerships. Anaesthesia 77:264–276. [↑](#footnote-ref-4)
