# Supplementary material for: Geographic barriers to care persist at the community healthcare level: Evidence from rural Madagascar
Source: PLOS Glob Public Health. 2022 Dec 27;2(12):e0001028. doi: 10.1371/journal.pgph.0001028 (PMC10022327; doi:10.1371/journal.pgph.0001028)
Supplement: S1 Data — (DOCX) [file pgph.0001028.s007.docx]

**FRENCH ABSTRACT/ RÉSUMÉ FRANÇAIS**

La distance géographique est un obstacle majeur à l'accès aux soins de santé, en particulier pour les communautés rurales disposant d'infrastructures de transport sous-développées et qui dépendent de moyens de transport non motorisés. Il existe un large consensus sur l'importance des agents de santé communautaires (ASC) pour réduire les effets de l'isolement géographique sur l'accès aux soins de santé. En raison du manque de données spatiales à échelle fine et de dossiers individuels de patients, nous savons peu de choses sur les effets précis des ASC sur l’amélioration des barrières géographiques à ce niveau du système de santé. En nous utilisant un ensemble de données participatives de haute qualité, qui comprend tous les chemins et bâtiments de la région, nous avons exploré l'impact de la distance géographique des sites d'ASCs sur l'utilisation des services des ASC pour les enfants de moins de 5 ans dans le district rural d'Ifanadiana, dans le sud-est de Madagascar, de 2018 à 2021. Nous avons ensuite utilisé cette analyse pour déterminer les caractéristiques clés d'une conception géographique optimale du système de santé communautaire, en optimisant spécifiquement un seul emplacement du site ou en installant des sites communautaires supplémentaires. Nous avons constaté que les taux de consultation par les ASC diminuaient avec l'augmentation de la distance parcourue par les patients jusqu'au site, d'environ 28,1 % par km. L'exercice d'optimisation a révélé que la majorité des sites ASC (50/80) étaient déjà dans un emplacement optimal ou partageaient un emplacement optimal avec une clinique de santé primaire. Nous prédisions que la relocalisation des sites ASC restants sur la base d'un optimum géographique unique n'augmentent les taux de consultation que de 7,4%. En revanche, l'ajout d'une deuxième site ASC était censé augmenter les taux de consultation de 31,5%, avec un effet plus important dans des régions le plus isolés. La distance géographique reste un obstacle au niveau de la santé communautaire, mais l'optimisation de l'emplacement du site ASC sur la base de la géographie seule n'entraînera pas de gains importants dans les taux de consultation. Il convient plutôt d'envisager des stratégies alternatives, telles que la création de sites ASC supplémentaires ou des soins proactifs.
