# Supplementary material for: Geographic barriers to care persist at the community healthcare level: Evidence from rural Madagascar
Source: PLOS Glob Public Health. 2022 Dec 27;2(12):e0001028. doi: 10.1371/journal.pgph.0001028 (PMC10022327; doi:10.1371/journal.pgph.0001028)
Supplement: Attachment — Submitted filename: chw-geog-response.pdf [file pgph.0001028.s008.pdf]

EDITOR COMMENTS:

- Please provide more details on the context of the study, including some general SES of the setting, population health indicators in particular children, and health delivery system.
- Please also provide a summary of evidence, if available, on the main barriers to access to PHC in the setting and justify why you have focused on geographic distance in your study.

Thank you for considering our manuscript for publication. In response to comments from the editor and reviewers, we have added additional information about the specific context of our study, including socio-demographic and health indicators and background about barriers to PHC in the district.

REVIEWIER 1:

Reviewer #1: This is an interesting study of public health importance, using an innovative approach to investigating access to community-based healthcare.

Thank you for your encouraging comment.

Line 92: As a stand-alone paper, a brief description of the health system in Madagascar would be helpful for readers who are not familiar with that country, to provide context. One should not have to look up previous publications from this project for this information. What is the total population of Madagascar and percentage of rural vs urban? What constitutes primary care in this region? How is the health system funded? Are patients required to pay for care and at what level? (see line 466 and reference to financial barriers to PHC, line 462 do wealthier people use private services rather than community services?). How does the referral system from CHW to PHC work? Also a sense of topography would be helpful – is Madagascar a hilly country, are there physical obstructions between rural services such as large rivers? What kind of non-motorised transport do people use to access services?

Thank you for this suggestion. We have added several additional paragraphs to describe the national and local context of this study (beginning line 135), in addition to a table of child health metrics (Table 1).

Line 502: What are the main factors affecting access to PHC in public sector facilities of Madagascar? It would be helpful to have these briefly described in the introduction and some justification for the focus on geographic distance. It is not sufficient to give references to other publications for this context.

We have added additional description of the barriers to access to PHC in public sector facilities (line 101):

> *Nationally, health professionals cite common barriers to healthcare access seen in other settings such as the cost of medicines, geographic distance, mistrust of the health system, and unsanitary conditions at health facilities (28). In addition, studies of healthcare access in rural districts of Madagascar have identified geographic distance to PHC as a key barrier to primary care, even when the health system is strengthened and financial barriers have been reduced (29,30).*

Line 97: it would be useful to see what the WHO recommended standards for workforce/population ratios and distances to the nearest health facility are. Does the term “medical professional” mean a doctor? If not, then the term health professional would be less confusing.

We have clarified our language here to refer to "medical doctors". We have also added some information about WHO recommended standards to allow the reader to understand these numbers in context. Unfortunately, there is no recommended standard for distance to the nearest health facility because the precise effect of distance on consultation rates and access is context-specific.

Line 99: it would be helpful to see what the child health indicators for Madagascar are, to get a sense of PHC use and context, for instance immunization coverage at age 5 years, infant and child mortality, chronic malnutrition ie stunting, and rates of childbirth at health facilities with skilled birth attendants (indicator of PHC coverage and neonatal health).

We have added this information to Table 1 for Madagascar and Ifanadiana district.

Line 112: “strong distance-decay effect on healthcare access” – how is this measured? These concepts may benefit from more explanation for readers who are not so familiar with this technique.

We have moved this sentence to discuss it in more detail under the Study Area heading. We have also clarified what this means: " Public health facilities provide over 80% of healthcare in the district, primarily at PHCs, but utilization rates decrease by 50% for every increase in travel time of 1 hour to the PHC (9)."

Line 180: CHW monthly reports - how reliable is this data? What checks were made to ensure that these data were trustworthy?

These data are collected as part of the regular health system reporting, but in Ifanadiana district CHWs are supported with additional supervision and review for data quality, ensuring data are trustworthy. We have added information about this to the text (line 222):

> *All CHWs in these fokontany receive additional training and supervision from Pivot, including training on data reporting methods. In addition, mobile teams provide regular supervision and trained staff evaluate subsets of data for accuracy These CHW reports, therefore, are a unique source of high quality data at the community level.*

Line 192: It is worth repeating the time period of the study as a reminder to the reader.

We have updated this sentence to include the time period.

Line 246: Is the tool available only in French? Is this the language used locally by CHW supervisors?

The tool is available in French and English and the application automatically selects the language based on the user's Operating System. All CHW supervisors speak French. We have added this detail to the manuscript.

Line 259: the reflexivity statement is well written, a good model and will be appreciated by readers.

Thank you! We found this to be a very fruitful exercise and it sparked a lot of additional discussion among the team.

Line 264: Is there any information on what the nature of these consultations were? Were all consultations captured or only those with children? What was the range? If the average

annual consultation rate was 1.73 consultations per capita for children only, this may be low. What was expected? Is there anything to compare with?

CHWs in Madagascar are only allowed to manage consultations for children under 5 years, and care is limited to the three diseases that are part of integrated community case management (iCCM): diarrhea, malaria, and acute respiratory infections. Our data is unfortunately aggregated into all-cause consultations, so it does not allow us to distinguish among these three diseases. We have added additional information about the consultations to the Methods and Results section (lines 154, 230, 312).

We agree that the consultation rate for children is low in Ifanadiana, and this was to be expected given the relative low performance of the health system via other health metrics (now provided in Table 1). In addition, other studies in this district have found similarly low consultation rates at all levels of the health system (Razafinjato et al. 2020 doi:10.1101/2020.12.11.20232611, Garchitorena et al. 2018 doi:10.1136/bmjgh-2018-000762, Rajaonarifara et al. 2022 doi:10.1136/bmjgh-2021-006824) . While this is indeed a result of our study, our focus is on the effect of distance on consultation rates and not evaluating tommunity healthcare more broadly. We therefore do not discuss this in depth in the Discussion section.

Line 292: “a decrease in annual consultation rates from 2.18 to 0.81 per capita” – where did 2.18 come from? The annual consultation rate per capita was given as 1.73 earlier.

Yes, we understand the confusion. The 1.73 figure is the overall average annual consultation rate in the district from the raw consultation rates. Here, we are describing the change in expected consultation rates due only to an increase in geographical isolation from 1km to 4km (assuming all other traits of that fokontany remained the same) as a counterfactual example for the reader based on the statistical model. We have rewritten this sentence to clarify this comparison: "For example, while keeping all other variables unchanged and at their mean value, an increase in geographic dispersion from 1 km to 4 km corresponds to a predicted decrease in annual consultation rates from 2.18 to 0.81 per capita." (line 248)

Line 488: “the increasing availability of these data” - how available is this data to most countries in Africa? Readers would be interested to know how to access systems that generate such data. It would be useful to be able to map a range of influential PHC factors such as access to safe water and sanitation facilities, roads, schools, local government offices, as well as clinics and personnel which also contribute to “spatial patterns in healthcare inequalities, identifying underserved communities” (line 489).

We certainly agree! It would be a huge benefit to many public health efforts to have more detailed information about the spatial location of transportation, medical, education, etc. infrastructures. The availability of this information has increased in recent years, but many of it is still fragmented and only available for certain sub-administrative districts, if at all.

For the systems that do exist on a large-scale, we have added additional detail about how to access them (556):

> *Several of these data sources are open-source and available online. For example, OpenStreetMap is an open-source dataset of transportation networks that can be viewed via a website(https://www.openstreetmap.org/) or downloaded via the Overpass API (https://overpass-turbo.eu/). In addition, Maina et al.* (63) *collated the location of over 98,000 public health facilities from 50 sub-Saharan African countries, which is available through the WHO's Global Malaria Programme (https://www.who.int/malaria/areas/surveillance/public-sector-health-facilities-ss-*

africa/en/). At the primary healthcare level, Weiss et al. (4) *estimated travel times to these health facilities.*

In conclusion, the paper needs to address more robustly the "so what?" question, what is the lesson for the PHC community? It seems obvious that more CHWs will be better than less, especially in dispersed areas of low population density (line 101:Two volunteer CHWs per fokontany, population of approx. 1200, but with a range in size from 1.5 to 3747 km2), the solution of distributing CHWs based on geographic equity rather than population numbers is well documented in the literature and apparently obvious to communities (see line 443: "communities are able to identify optimal locations without the use of a complex geographic algorithm).

It is difficult to get a sense from this paper of how well-served Madagascar is in PHC resources, including numbers of CHWs, than other countries in Africa. Countries with well-developed CHW programs usually involve CHWs who travel to households rather than being in static sites, as a principle of removing barriers of transport etc, but then need transport and other resources. They are also then more familiar with conditions that their patients come from.

We have added additional information about the health system in Madagascar to the Methods section to help contextualize these results. We have also added information regarding the new community healthcare recommendations release by the MMoPH in the past several months, which includes a prioritization of households visits, but which has not yet been implemented (line 151).

> *Community health care, including diagnoses and distribution of limited medicines, takes place at the community health site of each fokontany, a static location, although more recent policies (2023 onward) recommend home visits.*

It would be interesting to unpack the gender dynamics more – do the communities think male CHWs are doctors (a phenomenon in many countries) and therefore consult them more? Do women feel satisfied consulting male CHW for pregnancy related care? This may be an area for further research.

Yes, we agree this is an important avenue for future research. We direct the reader to several studies that more directly consider the gender dynamics of CHWs in our citations. Unfortunately, we believe this to be out of the scope of the current study, which focuses on geographic barriers at the community level. As we are familiar with some of the work on gender dynamics, we included this variable to control for any confounding effects, but we believe a more in-depth, purposefully designed study would be needed to address this question properly in Ifanadiana. We have added a note that this would be a useful future study (line 545).

To persuade readers of the usefulness of this technique of measuring geographic access to PHC, rather than confirming the obvious, the authors could present a more detailed picture of PHC in Madagascar into which this new knowledge creates a better understanding of improving access to care (an indicator of quality of care).

Yes, we agree and there have certainly been many studies on geographic access to primary health clinics and we mention this in the manuscript (line 475). Geographic access to community health workers, on the other hand, has been less studied. While it may seem obvious that geographic barriers would persist at distances less than 5km, CHW are installed specifically to mediate those barriers, based on an assumption that a geographical distance of such a small size would not impact health-seeking behaviors. Indeed, thresholds for health system coverage are often 30 minutes in settings where motorized transportation

is common or one hour where non-motorized transport is the norm. We find that, even within this threshold, geographic barriers persist. We have expanded on these points in the manuscript (line 475):

> *This trend is not unique to Madagascar, and negative impacts of geographic accessibility on primary and secondary healthcare access are well studied (see Guagliardo (52) for a review). The findings of this study go one step further and show that geographic isolation remains a barrier for CHW access, where distances to seek care are much smaller. This contributes to a nascent, but growing literature studying the geography of community healthcare (53,54), with the general consensus that geographic barriers also exist at this much finer spatial scale. Often, a threshold of 5km distance or 1 hour travel time is used to define spatial coverage of healthcare access (e.g.* (4)*, but we find that, for community care, a meaningful effect of distance on healthcare utilization remains even within that definition of "coverage".*

In addition, as the reviewer mentions, testing for evidence of this barrier in Madagascar specifically not only adds to our general scientific consensus by testing a theory in a new geographic location, but provides further evidence that alternative approaches to community healthcare, such as pro-CCM or home visits, are needed in Madagascar. This is particularly important given the current national policy which prohibits paying CHWs a salary to carry out such work.

We have added discussion of what our results mean specifically for Madagascar health policy to the manuscript (line 507):

> *Our results have important implications for the design of national community health programs in Madagascar and elsewhere. The community health policy of Madagascar in recent years required two CHWs per fokontany regardless of their size or geographic dispersion of the population. In late 2022, national community health guidelines were updated to recommend one CHW for every 300 households in a fokontany (36), although this policy has not yet been implemented. However, the guidelines do not include recommendations for the distribution of CHWs or health sites with respect to spatial patterns in population densities or transport networks. Our results suggest that in addition to the recommended ratios, the location of CHWs should be adapted to the local geography of CHW catchments, and additional interventions may be necessary to reach isolated communities. Our results also support the need for proactive community case management programs (pro-CCM), which have been implemented elsewhere (55), and were recently recommended in the MMoPH's community health policy. Under pro-CCM, CHWs visit each household within a catchment at regular intervals to provide basic services and conduct educational campaigns in addition to other elements, such as cost-free care, CHW professionalization and case follow-up regimes (56,57). Given the strong association between high geographical dispersion and low consultation rates, the implementation of pro-CCM programs in Madagascar has great potential for improving health outcomes.*

REVIEWER 2:
General comment:
This is a valuable and important paper on a topic of great importance that has received little attention in the peer-reviewed literature – namely the optimal geographic location of CHW and geographic influences on CHW utilization. It is simplistically assumed that geographic barriers to access care are removed when services are provided by CHWs, and this paper makes the important point that this is not necessarily the care, The authors are to be

congratulated on this useful piece of research. The paper is well-written and well-organized.

Specific comments:
1. The analysis employs a lot of sophisticated geographic analyses that I am not qualified to comment on. On the surface, it looks like it has been carefully done.

Thank you.

2. It seems to me that the authors should raise the possibility of having the government assign more than 2 CHWs in those fokotanys that are larger in size and that have larger populations. This should be mentioned in the Discussion section, providing more information on the size and population variability among fokotanys would be useful for the Results section. Why not propose a more optimal model that doesn't limit each fokotonay to just 2 CHW sites.

Yes, we would expect that the addition of even more CHW sites would reduce the impact of geographical isolation on community healthcare even further. However, this problem of optimization is even more complex than that of geographical optimization, as it would involve estimating a cost-benefit for the addition of each additional CHW. Without a cost to limit the total number of CHW sites, the optimization algorithm would simply place a site in each residential area, thereby reducing geographic isolation as much as possible. This type of cost-benefit analysis is an interesting health economics question, but we believe outside the scope of our study, which is focused focused primarily on geographic access and potential solutions given current resources in Madagascar.

At the initial time of submission, the potential for adding additional CHW to a fokontany was unlikely given the current national policy, which is why we did not explore this. However, the community health guidance has since been updated and does include a recommendation to increase the number of CHWs based on the population of a fokontany. It does not, however, mention an increase in the number of sites or the spatial distribution of sites relative to the spatial distribution of the population. We believe our study contributes to these newly adopted policies by demonstrating how the addition of just one more site, placed in an optimal location, could increase consultation rates while limiting the number of additional CHWs needed. We have added additional discussion in the light of this new policy (line 487):

> *In late 2022, national community health guidelines were updated to recommend one CHW for every 300 households in a fokontany (36), although this policy has not yet been implemented. However, the guidelines do not include recommendations for the distribution of CHWs or health sites with respect to spatial patterns in population densities or transport networks. Our results suggest that in addition to the recommended ratios, the location of CHWs should be adapted to the local geography of CHW catchments, and additional interventions may be necessary to reach isolated communities.*

Also, it would be interesting to add a table that lists the number of fokotanys by category or size and population.

We have added a table of the fokontany sizes in the district of Ifanadiana to the supplement (Table S1).

3. Likewise, the Discussion section should also raise the possibility of relying more on home visits from CHWs rather than simply expecting the beneficiaries to come to the CHW sites. The authors briefly mention in the Discussion section the power of Pro-CCM, but the advantages of home visitation more broadly could be emphasized and strengthened

beginning on line 414. It is good to see that you are testing this out in a pilot area! Here are some references that support that:
Johnson, A. D., Thiero, O., Whidden, C., Poudiougou, B., Diakite, D., Traore, F., . . . Kayentao, K. (2018). Proactive community case management and child survival in periurban Mali. BMJ Glob Health, 3(2), e000634. doi:10.1136/bmjgh-2017-000634
Perry, H. B., Rassekh, B. M., Gupta, S., & Freeman, P. A. (2017). Comprehensive review of the evidence regarding the effectiveness of community-based primary health care in improving maternal, neonatal and child health: 7. shared characteristics of projects with evidence of long-term mortality impact. J Glob Health, 7(1), 010907. doi:10.7189/jogh.07.010907

Thank you for these citations. We definitely agree regarding the benefits of proactive care and home visits even without the larger umbrella of pro-CCM. We have added some additional language about the potential for this alternative program to mitigate the negative effect of geographic isolation (lines 495):

> *Our results also support the need for proactive community case management programs (pro-CCM), which have been implemented elsewhere* (55)*, and were recently recommended in the MMoPH's community health policy.* Under pro-CCM, CHWs visit each household within a catchment at regular intervals to provide basic services and conduct educational campaigns in addition to other elements, such as cost-free care, CHW professionalization and case follow-up *regimes (56,57). Given the strong association between high geographical dispersion and low consultation rates, the implementation of pro-CCM programs in Madagascar has great potential for improving health outcomes.*

4. On line 67, another appropriate reference to cite there is:
Perry, H. (2020). Health for the People: National Community Health Programs from Afghanistan to Zimbabwe. Retrieved from https://pdf.usaid.gov/pdf_ and https://chwcentral.org/wp-

Thank you for the citation, it is a great resource for community health. We have added it to the text.

5. On line 172, how were the clusters selected?

We have added additional information on the sampling scheme (lines 203):

> *The sampling scheme was a two-stage cluster sampling design, where 40 clusters were sampled at random within each of two strata, within the Pivot initial catchment and the rest of the district. Twenty households per cluster were then randomly selected to be surveyed. The IHOPE cohort was implemented by the Madagascar National Institute of Statistics and was based primarily on the internationally validated Demographic and Health Surveys (DHS). Further details on the longitudinal survey can be found in Miller et al. (37) and Ezran et al. (39).*
